# Supplementary material for: Single-electron charge sensing in self-assembled quantum dots
Source: Sci Rep. 2018 Sep 18;8:13188. doi: 10.1038/s41598-018-31268-x (PMC6143615; doi:10.1038/s41598-018-31268-x)
Supplement: Supplementary file 1 — Supplementary Information [file 41598_2018_31268_MOESM1_ESM.pdf]

Supplementary information

Single-electron charge sensing in self-assembled quantum dots

Haruki Kiyama[1], Alexander Korsch[2], Naomi Nagai[3], Yasushi Kanai[1], Kazuhiko Matsumoto[1], Kazuhiko Hirakawa[3], Akira Oiwa[1,4]

[1] *The Institute of Scientific and Industrial Research, Osaka University, 8-1, Mihogaoka, Ibaraki, Osaka 567-0047, Japan*

[2] *Lehrstuhl für Angewandte Festkörperphysik, Ruhr-Universität Bochum, Universitätsstraße 150, Gebäude NB, D-44780 Bochum, Germany*

[3] *Institute of Industrial Science, the University of Tokyo, 4-6-1 Komaba, Meguro, Tokyo 153-8505, Japan*

[4] *Center for Spintronics Research Network, Graduate School of Engineering Science, Osaka University, 1-3 Machikaneyama, Toyonaka, Osaka 560-0043, Japan*

E-mail: kiyama@sanken.osaka-u.ac.jp

Estimation of the lever-arm factor for a quantum dot weakly tunnel-coupled to reservoirs

In Fig. 3(c) in the main text, we show the N+1 state fraction of the quantum dot QD_2 in sample A as a function of the dot chemical potential μ_{QD2}. Although the current through QD_2, I_2, is too small to measure at the real-time charge sensing condition, we estimate the lever-arm factor of the side-gate voltage V_{SG2L} to μ_{QD2} from the charge sensing measurement of QD_2. Figures S1(a) to (c) show the current through QD_1, I_1, as a function of side-gate voltages V_{SG1} and V_{SG2L} at different source and drain bias voltages across QD_2, V_{SD2}. The gate voltage conditions are slightly changed from those in Fig. 3(a) supposedly because of the charge redistribution around the QDs. The Coulomb peak resonance in QD_2 appears at the shift of the I_1 Coulomb peak as indicated by the red arrows. As V_{SD2} decreases, the V_{SG2L} value of the QD_2 resonance decreases without significant broadening of the resonance. This indicates that the source electrode has larger tunnel coupling with QD_2 than the drain electrode, and that electron tunnelling occurs dominantly between QD_2 and the source electrode. Note that V_{SG1} is slightly increased with decreasing V_{SD2} to tune the chemical potential of QD_1, which hardly changes μ_{QD2}. Figure S2 shows the V_{SG2L} value for the QD_2 resonance as a function of V_{SD2}. From a linear fit to this data, we estimate the lever-arm factor of V_{SG2L} to μ_{QD2} to be 21 ± 1 meV/V by assuming that the source and the drain electrode have almost the same capacitance with QD_2.

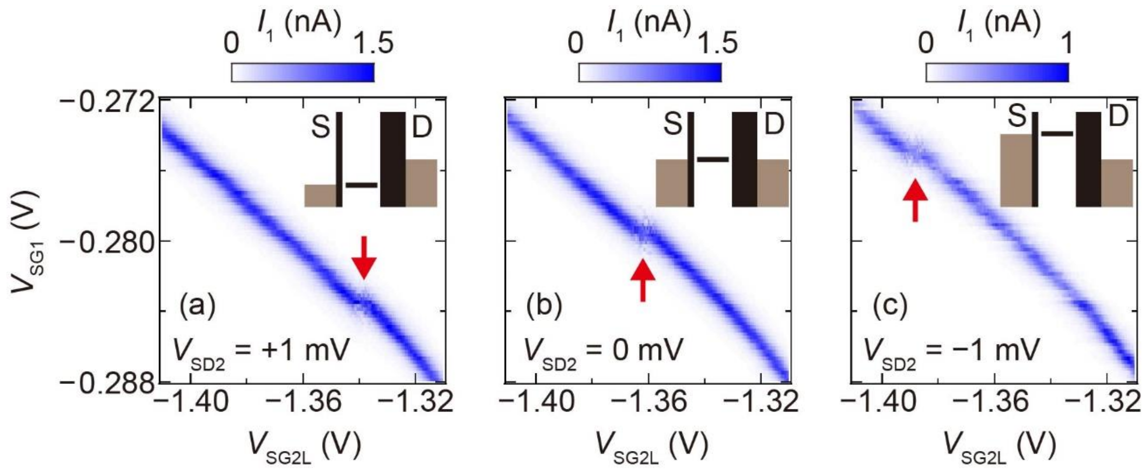

Figure S1. Intensity plot of I_1 in sample B as a function of $V_{SG1} = V_{SG1L} = V_{SG1R}$ and V_{SG2L} at (a) V_{SD2} = +1 mV, (b) V_{SD2} = 0 mV, and (c) V_{SD2} = −1 mV. The back-gate voltage is V_{BG} = −0.5 V, and the source and drain bias voltage across QD_1 is V_{SD1} = 70 μV. Insets schematically illustrate the energy diagram at the Coulomb peak resonance in QD_2 for each V_{SD2}.

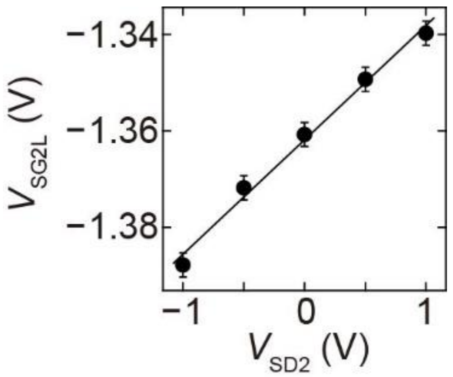

Figure S2. V_{SG2L} values for the QD_2 resonance as a function of V_{SD2}. The solid line represents a linear fit to the data.
